# Molecular and Functional Analyses of the Primordial Costimulatory Molecule CD80/86 and Its Receptors CD28 and CD152 (CTLA-4) in a Teleost Fish

**DOI:** 10.3389/fimmu.2022.885005

**Published:** 2022-06-16

**Authors:** Tao-Zhen Lu, Xun Liu, Chang-Song Wu, Zi-You Ma, Yang Wang, Yong-An Zhang, Xu-Jie Zhang

**Affiliations:** ^1^ State Key Laboratory of Agricultural Microbiology, College of Fisheries, Huazhong Agricultural University, Wuhan, China; ^2^ Engineering Research Center of Green Development for Conventional Aquatic Biological Industry in the Yangtze River Economic Belt, Ministry of Education, Wuhan, China; ^3^ Guangdong Provincial Key Laboratory of Pathogenic Biology and Epidemiology for Aquatic Economic Animals, Guangdong Ocean University, Zhanjiang, China; ^4^ Hubei Hongshan Laboratory, Huazhong Agricultural University, Wuhan, China; ^5^ Laboratory for Marine Biology and Biotechnology, Pilot National Laboratory for Marine Science and Technology, Qingdao, China

**Keywords:** CD80/86, CD28, CD152, grass carp, T cell activation

## Abstract

The moderate activation of T cells in mammals requires the costimulatory molecules, CD80 and CD86, on antigen-presenting cells to interact with their respective T cell receptors, CD28 and CD152 (CTLA-4), to promote costimulatory signals. In contrast, teleost fish (except salmonids) only possess CD80/86 as their sole primordial costimulatory molecule. However, the mechanism, which underlies the interaction between CD80/86 and its receptors CD28 and CD152 still requires elucidation. In this study, we cloned and identified the *CD80/86*, *CD28*, and *CD152* genes of the grass carp (*Ctenopharyngodon idella*). The mRNA expression analysis showed that *CD80/86*, *CD28*, and *CD152* were constitutively expressed in various tissues. Further analysis revealed that *CD80/86* was highly expressed in IgM^+^ B cells. Conversely, *CD28* and *CD152* were highly expressed in CD4^+^ and CD8^+^ T cells. Subcellular localization illustrated that CD80/86, CD28, and CD152 are all located on the cell membrane. A yeast two-hybrid assay exhibited that CD80/86 can bind with both CD28 and CD152. *In vivo* assay showed that the expression of *CD80/86* was rapidly upregulated in *Aeromonas hydrophila* infected fish compared to the control fish. However, the expression of *CD28* and *CD152* presented the inverse trend, suggesting that teleost fish may regulate T cell activation through the differential expression of CD28 and CD152. Importantly, we discovered that T cells were more likely to be activated by *A. hydrophila* after CD152 was blocked by anti-CD152 antibodies. This suggests that the teleost CD152 is an inhibitory receptor of T cell activation, which is similar to the mammalian CD152. Overall, this study begins to define the interaction feature between primordial CD80/86 and its receptors CD28 and CD152 in teleost fish, alongside providing a cross-species understanding of the evolution of the costimulatory signals throughout vertebrates.

## Introduction

It is well established that the optimal activation of naive T cells in mammals requires both TCR and costimulatory signals. The costimulatory signals are predominantly produced by the interaction between costimulatory molecules CD80 and CD86 on antigen-presenting cells (APCs) and their receptors CD28 and CD152 (CTLA-4) on T cells (1–3). Mammalian type I membrane glycoproteins, CD80 and CD86, are closely located on the same chromosome and possess similar genomic organizations and domain structures. CD80 and CD86 both consist of a signal peptide, two extracellular Ig-like domains (IgV and IgC), a transmembrane region and a cytoplasmic domain (4). CD28 and CD152 both are members of the immunoglobulin (Ig) superfamily containing a signal peptide, an Ig-like domain, a transmembrane region, and a cytoplasmic domain (2). Though both CD80 and CD86 can bind to CD28 and CD152, the main ligand for CD152 is CD80, while CD86 is the main ligand for CD28. Moreover, CD80 and CD86 (mainly CD86) augment T cell activation through initially binding to CD28. However, the over-activation of T cells is inhibited through a higher affinity binding by mainly CD80 to CD152 (5–10).

In contrast to the mammalian pathway, previous studies have revealed that teleost species, except salmonids, only possess a single primordial CD80/86 molecule, including zebrafish (*Danio rerio*), orange-spotted grouper (*Epinephelus coioides*), Nile tilapia (*Oreochromis niloticus*), and yellow catfish (*Pelteobagrus fulvidraco*) (11–15). The functional analysis in zebrafish suggested that the teleost CD80/86 is similar to the mammalian CD86 than the CD80. The costimulatory receptor CD28 has been identified in several teleost species, such as the Nile tilapia, zebrafish, rainbow trout (*Oncorhynchus mykiss*), rock beam (*Oplegnathus fasciatus*), sea bass (*Dicentrarchus labrax*), tongue sole (*Cynoglossus semilaevis*), pufferfish (*Takifugu rubripes*), and Japanese flounder (*Paralichthys olivaceus)* (16–22). However, the costimulatory receptor CD152 has only been identified in sea bass and rainbow trout. Presently, the mechanism underlying the primordial CD80/86 interaction with its CD28 and CD152 receptors is still unknown.

In this study, we cloned and identified the *CD80/86*, *CD28*, and *CD152* genes from grass carp (*Ctenopharyngodon idella*). The binding activities of CD80/86 in grass carp with the receptors CD28 and CD152 were confirmed. Further analysis revealed that the teleost fish may regulate T cell activation through the differential expression of CD28 and CD152. Further, as in mammals, the grass carp CD152 may also be an inhibitory receptor of T cell activation. This study provides preliminary data for the interaction feature between primordial CD80/86 and its CD28 and CD152 receptors in teleost fish. Additionally, it specifies new insights into the evolution of the costimulatory signals in vertebrates.

## Materials and Methods

### Fish and Bacteria

Healthy grass carp (weighing 200 ± 20 grams) were purchased from a fish farm in Xiantao, China. For at least two weeks prior to the experimental analysis, the grass carp were acclimatized and maintained in the laboratory fish culture system with a water control device. All animal experiments were approved by the Committee on the Ethics of Animal Experiments at Huazhong Agricultural University.

*Aeromonas hydrophila* XS91-4-1 (23), which is a virulent strain isolated from fish, was used in the infection experiment.

### Cloning of CD80/86, CD28, and CD152 Genes in Grass Carp

The high-quality grass carp genome was re-sequenced and assembled by our laboratory, which has been deposited under NCBI BioProjects with the accession number: PRJNA745929 (24). The grass carp *CD80/86*, *CD28*, and *CD152* genes were searched using the Basic Local Alignment Search Tool (BLAST). To ensure verification of *CD80/86*, *CD28*, and *CD152*, the gene coding sequences (CDS) for these genes were amplified by PCR. Initially, the total RNA was extracted from the head kidney of the grass carp using TRIzol Reagent (Takara). The first-strand cDNA was synthesized using a PrimeScript™ First-Strand cDNA Synthesis Kit (Takara). Specific primers were designed to bind to the 5’ or 3’ untranslated regions (UTR) of *CD80/86*, *CD28*, and *CD152* (**Table 1**). Following this, the CDS of these genes was amplified from the cDNA. The PCRs were performed as a 50 μL reaction, which contained a prime star mix (25 μL), a forward primer (10 μM; 2 μL), a reverse primer (10 μM; 2 μL), cDNA (1 μL), and DEPC H_2_O (20 μL). The amplification program was 95°C for 3 minutes, followed by 35 cycles of 95°C for 15 seconds, 58°C for 30 seconds, and 72°C for 30 seconds. The PCR products were electrophoresed on a 1.5% agarose gel, following which the bands were extracted using a HiPure Gel Pure DNA Mini Kit (Magen) and sequenced by Tsingke Biotechnology Co., Ltd.

**Table 1:** Primers used in this study.

Name	Sequence (5’→3’)	Application
pEGFP-N1-CD80/86-F	CCGGAATTCGCATGAATTTAACGCTGCTCTG	Prokaryotic expression
pEGFP-N1-CD80/86-R	CGCGGATCCGCGACGCTCTTGAGCTGTAAATGG	
pEGFP-N1-CD28-F	CCGGAATTCGCATGGAGATTTTCAGGACATCTG	
pEGFP-N1-CD28-R	CGCGGATCCGCTACTACAGTGTCTTTGCGAAAAC	
pEGFP-N1-CD152-F	CCGGAATTCGCATGATTATCACACTTATTAGAAT	
pEGFP-N1-CD152-R	CGCGGATCCGCGAATTTCATATAGCTCTCTTTT	
pBT3-SUC-CD80/86-F	TGCAATGGCCATTACGGCCATGACAGATGATGACAG	Yeast two hybrid assay
pBT3-SUC-CD80/86-R	GCAGATGGCCATTACGGCCGACGCTCTTGAGCTGTAAATGGCTCTTGAGCTGT	
pPR3-CD28-F	CGCGGATCCATGCAGAGCATATGCAAAGGTATGCAAAGG	
pPR3-CD28-R	CCGGAATTCTCATACTACAGTGTCTTTGCTGTCTTTGC	
pPR3-CD152-F	CGCGGATCCATGCTGCGTGTGTCTCAGCCTGTCTCAGCC	
pPR3-CD152-R	CCGGAATTCCTAGAATTTCATATAGCTCTCTATAGCTCTC	
CD80/86-F	GCATGAATTTAACGCTGCTCTG	Gene cloning
CD80/86-R	GCGACGCTCTTGAGCTGTAAATGG	
CD28-F	GCATGGAGATTTTCAGGACATCTG	
CD28-R	GCTACTACAGTGTCTTTGCGAAAAC	
CD152-F	GCATGATTATCACACTTATTAGAAT	
CD152-R	GCGAATTTCATATAGCTCTCTTTT	
β-actin-QF	AGCCATCCTTCTTGGGTATG	Quantitative real-time PCR
β-actin-QR	GGTGGGGCGATGATCTTGAT	
CD80/86-QF	GGACCATCAACCAAACCAT	
CD80/86-QR	AACACAAGCACGACGACAA	
CD28-QF	ACGAAACCTGCCCAAAACAA	
CD28-QR	TGCCAGCAAGGCTAATAATA	
CD152-QF	GAGGGGAAGACACAGACCTC	
CD152-QR	AACAACAGTGCCGTTCCCAA	
LCK-QF	CGCAGAGGGAATGGCTTACA	
LCK-QR	AGTTTATGGCCTCAGGTGCC	
CD154-QF	CGTCGGGCTTAACAGCAAAA	
CD154-QR	ACCAGGGAGGCTGTACACAA	
CD4-1-QF	AGATGTGTCCAGGTGTCATAGT	
CD4-1-QR	TGGAATTTTGACTGTATAGGATGA	
CD4-2-QF	CACTGCGACATTACAGGACAC	
CD4-2-QR	GCCTCCTTCAGAAACTTCAACA	
CD8-QF	GGAGTCTCTGCACGGATCTAT	
CD8-QR	GTGTAGTGTTCCGAATTTAAGTC	
CD69-QF	CAACATGAACGACACGAACGA	
CD69-QR	ACCTGAAGACCACTGCCATTT	
16s-QF	GAGATACGGGAGTGCCTTCG	
16s-QR	GTGCTGGCAACAAAGGACAG	

The restriction enzyme sites are underlined. F, forward; R, reverse.

### Bioinformatic Analysis of Grass Carp CD80/86, CD28, and CD152 Genes

The signal peptide, transmembrane region, and functional domains of CD80/86, CD28, and CD152 were predicted using SignalP 5.0 Server (https://services.healthtech.dtu.dk/service.php?SignalP-5.0), TMHMM Server 2.0 (https://services.healthtech.dtu.dk/service.php?TMHMM-2.0), and SMART (http://smart.embl.de/), respectively. The N-linked glycosylation sites in CD80/86, CD28, and CD152 were predicted using NetNGlyc 1.0 Server (25). The *CD80/86, CD28*, and *CD152* gene organizations, including exon, intron, and UTR were determined by aligning the cDNA and gene sequences. Multiple sequence alignment was conducted by the ClustalX program (version 3.0) using the amino acid (aa) sequences. Phylogenetic analysis was conducted according to the amino acid sequences using the neighbor-joining method bootstrapped 1000 times with the MEGA 4.1 software.

### Detection of the mRNA Expression of *CD80/86*, *CD28*, and *CD152* Genes in Grass Carp Tissues

Initially, the healthy grass carp were anesthetized with MS-222. The blood was subsequently extracted from the caudal vein, while any remaining blood was removed by cardiac perfusion using phosphate-buffered saline (PBS; pH 7.4; Gibco). The thymus, head kidney, trunk kidney, spleen, gill, intestine, skin, liver, heart, muscle, and brain were also harvested. The total RNA was extracted from the samples using TRIzol Reagent (Takara), and the primary cDNA strand was synthesized using a PrimeScript™ First-Strand cDNA Synthesis Kit (Takara).

The *CD80/86*, *CD28*, and *CD152* mRNA expression levels in the grass carp tissues were measured by quantitative real-time PCR (qPCR) in a CFX Connect™ Real-Time System (Bio-Rad) with the primers pairs listed in **Table 1**. The *β-actin* gene was used as the internal control. The qPCR was performed in a 20 µL reaction volume containing 1 µL of each primer (10 mM), 1 µL of cDNA, 10 µL of ChamQ Universal SYBR qPCR Master Mix (Vazyme), and 7 µL of PCR-grade water. The amplification program was: 94°C for 5 minutes followed by 40 cycles at 94°C for 10 seconds and 60°C for 1 minute. Melt curve analysis of the amplification products was performed from 70°C to 95°C at the end of each reaction to confirm the generation of a single product. The *CD80/86*, *CD28*, and *CD152* gene expression levels were determined by the cycle threshold (Ct) using the 2^−ΔCt^ method.

### Isolation of Leukocytes

Leukocytes were isolated from the head kidney (HKLs) of grass carp as previously described (26). Briefly, the head kidney tissue was dissociated into cell suspensions with Dulbecco’s Modified Eagle Medium (DMEM; Invitrogen), before being passed through a 100 µm cell strainer (Biosharp). The cell suspensions were carefully layered onto a 51%/34% discontinuous Percoll density gradient (GE Healthcare) and centrifuged at 400 × *g* for 30 minutes at 4°C. The leukocytes laying at the interface were then collected, washed, and re-suspended in PBS supplemented with 2% fetal bovine serum (FBS; Gibco).

### Detection of the *CD80/86*, *CD28*, and *CD152* Genes mRNA Expression in Grass Carp Leukocyte Subpopulations

HKLs were isolated and stained with mouse anti-grass carp IgM monoclonal antibodies (mAbs), rat anti-carp CD4 mAbs (Cosmo Bio), and rat anti-carp CD8 mAbs (Cosmo Bio) respectively to detect the mRNA expression of grass carp *CD80/86*, *CD28*, *CD152, CD4-1, CD4-2*, and *CD8* genes in leukocyte subpopulations, as previously described (27–31). The APC-goat anti-mouse IgG (Biolegend) and APC-goat anti-rat IgG (Biolegend) Abs were used as the secondary Abs. The IgM^+^ B cells, IgM^-^ lymphocytes (containing CD4^+^ T cells, CD8^+^ T cells, and IgT^+^ B cells) (26–28), myeloid I subpopulation cells (containing monocytes/macrophages and plasma cells) (27–29), and myeloid II subpopulation cells (containing granulocytes) (32) were sorted from HKLs stained with IgM mAbs using a fluorescence-activated cell sorter (FACS; FACSAria™ III, BD Biosciences). The CD4^+^ T cells or CD8^+^ T cells, CD4^-^ lymphocytes (containing CD8^+^ T cells and B cells) or CD8^-^ lymphocytes (containing CD4^+^ T cells and B cells), myeloid I subpopulation cells, and myeloid II subpopulation cells were sorted from HKLs stained with CD4 or CD8 mAbs. The total RNA was extracted from the sorted cells using a RNeasy Mini Kit (MAGEN) and the cDNA was synthesized using Hiscript III Reverse Transcriptase (Vazyme). The *CD80/86*, *CD28*, *CD152, CD4-1, CD4-2 and CD8* genes mRNA expression levels in the sorted cell populations were determined by qPCR using the previously described method (28, 29), while *β-actin* was used as the internal control.

### Subcellular Localization Assay for CD80/86, CD28, and CD152 in Grass Carp

The CDS of *CD80/86*, *CD28*, and *CD152* were amplified by PCR using specifically designed primers (**Table 1**). The PCR products were digested and inserted into a pEGFP-N1 plasmid using the EcoR I and BamHI restriction enzymes. The pEGFP-CD80/86, pEGFP-CD28, and pEGFP-CD152 plasmids were transformed into Trans5*α* chemically competent cells (TransGen), which were subsequently cultured in LB Broth supplemented with 50 mg/L kanamycin for 12 hours at 37°C. The plasmids were then extracted from the cells using an E.Z.N.A. Plasmid Maxi Kit (Omega). HEK293T cells (5 × 10^5^ cells) were seeded into each well of a 12-well plate (VWR) and cultured in DMEM (Gibco) supplemented with 10% FBS (Gibco) for 12 hours at 37°C in 5% CO_2_. Then, either 1.6 µg of the pEGFP-N1, pEGFP-CD80/86, pEGFP-CD28, or pEGFP-CD152 plasmid was transfected into the HEK293T cells in each well using 4 µL TransIntro™ EL Transfection Reagent (TransGen), according to the manufacturer’s instructions. At 24 hours post-transfection, 4% paraformaldehyde (Biosharp) was added to the cells for 15 minutes to fix them. Afterward, the cells were stained with 100 ng/mL DAPI (Biosharp) for 15 minutes at room temperature. Finally, confocal microscopy (Nikon N-STORM) was used to obtain fluorescence images of the transfected HEK293T cells.

### Detection of the Binding Activity of CD80/86 to CD28 and CD152 Using a Yeast Two-Hybrid Assay

A yeast two-hybrid assay was performed to detect the possible interaction between CD80/86 and CD28 or CD152 in grass carp (33–35). Firstly, the CDS (without the signal peptide-coding region) of *CD80/86* was amplified (primers listed in **Table 1**) and subcloned into the pBT3-SUC plasmid, which was used as the bait vector. The CDS (without the signal peptide-coding region) of *CD28* and *CD152* were amplified and subcloned into the pPR3-N plasmid, which was used as the prey vector. To test if the recombinant plasmids were successfully cloned into the strain, the two different kinds of plasmids in each group were co-transformed into NMY51 yeast strain and selected for on a synthetic double-dropout medium (DDO: SD/-Trp-Leu) following 3 days at 30°C. The positive clones were selected on a triple dropout medium (TDO: SD/−Leu/−Trp/−His) supplemented with 5 mM 3-Aminotriazole (3-AT) and a quadruple dropout medium (QDO: SD/−Leu/−Trp/−His/−Ade) following 4-5 days at 30°C. The 3-AT was added to inhibit the growth of false-positive clones. For self-activation detection, the pBT3-SUC-CD80/86 and pPR3-N plasmids were co-transformed into the competent NMY51 yeast cells. Moreover, both pBT3-SUC-CD80/86 and pOst1-NubI were co-transformed into the competent NMY51 yeast cells to operate as a positive functional control. Additionally, the pTSU2-APP and pNubG-Fe65 plasmids were co-transformed into competent NMY51 yeast cells as the positive control, while the pTSU2-APP and pPR3-N plasmids were likewise co-transformed as the negative control. Finally, pBT3-SUC-CD80/86 was co-transformed into the competent NMY51 yeast cells with either pPR3-N-CD28 or pPR3-N-CD152 to detect the binding activity of CD80/86 to CD28 and CD152.

### Infection of Grass Carp by *A. hydrophila*

To detect the immune responses of CD80/86, CD28, and CD152 during an infection, the grass carp were intraperitoneally injected with 100 µL of *A. hydrophila*, which had been suspended in PBS (1×10^7^ CFU/mL). Conversely, the control fish were injected with only 100 µL of PBS. Following, the grass carp were anesthetized with MS-222 at time points of 0-, 6-, 12-, 24-, 48-, and 72-hours post-injection (hpi), and the head kidneys and spleens were sampled. The total RNA was extracted, and the cDNA was synthesized as described above. Following qPCR, the *CD80/86*, *CD28*, *CD152*, *CD4-1*, *CD4-2*, and *CD8* expression levels in *A. hydrophila*-infected fish were compared to the control fish using the 2^−ΔΔCt^ method, with *β-actin* as the internal control. Besides, the expression level of *A. hydrophila* 16s RNA in head kidney and spleen of infected fish was detected by qPCR to indicate the tissue bacterial load using the 2^−ΔCt^ method, with grass carp *β-actin* as the internal control.

### Production of Specific Polyclonal Abs (pAbs) Against CD152 in Grass Carp

The antigen peptide (RVDMIITGLRGEDTD) of CD152 in grass carp was synthesized by GenScript Ltd. The specific pAbs were produced using the CD152 peptide (AtaGenix Ltd). Briefly, the CD152 peptide was coupled to a keyhole limpet hemocyanin (KLH) using the 1-ethyl-3-(3-dimethylaminopropyl) carbodiimide (EDC)/*N*-hydroxysuccinimide (NHS) coupling method and used to raise pAbs in rabbits. After the IgG in the antiserum was purified by a HiTrap protein G column, the CD152 peptide-specific pAbs were further purified using CD152 peptide coupled cyanogen bromide (CNBr)-activated Sepharose through affinity chromatography.

The specificity of the rabbit anti-CD152 pAbs was tested by western blot. Briefly, the membrane proteins of HKLs (5×10^6^ cells) were extracted using Mem-PER Plus Membrane Protein Extraction Kit (Thermo). The samples were electrophoresed on 12% Tris-HCl BeyoGel™ Plus Precast PAGE Gel (Beyotime) under non-reducing conditions and transferred to a nitrocellulose blotting membrane (Pall). The membrane was blocked for 1 h at room temperature in TBST buffer (25 mM Tris˗HCl, 150 mM NaCl, 0.1% Tween 20, pH 7.5) containing 5% nonfat dry milk, then incubated with rabbit anti-grass carp CD152 pAbs (1:1000 diluted with TBST) at 4°C overnight. After three washes with TBST, the membrane was incubated with HRP-conjugated goat anti-rabbit IgG (Thermo; 1:5000 diluted with TBST) for 1 h at room temperature. After additional three washes with TBST, the membrane was stained with Clarity Western ECL Substrate (Bio-Rad) and scanned using the Amersham Imager 600 (GE Healthcare).

A blocking experiment on the anti-CD152 pAbs was also performed to verify the specificity of the rabbit anti-CD152 pAbs, which used the CD152 peptide as previously described (36). The rabbit anti-CD152 pAb was pre-incubated with the CD152 peptide at molar ratios of 1:0, 1:10, 1:50, 1:100, and 1:150 respectively for 45 minutes, prior to the HKL staining for flow cytometry analysis. Moreover, cell sorting was used to further verify the specificity of the rabbit anti-CD152, as previously described (27). The HKLs were stained with rabbit anti-CD152 pAbs, prior to staining with the FITC-goat anti-rabbit IgG (Abclonal) as above described. Finally, the CD152^+^ and CD152^-^ lymphocytes were sorted using a fluorescence-activated cell sorter (FACS; FACSAria™ III, BD Biosciences).

### Blocking CD152 by Anti-CD152 pAbs

To further clarify the immune role of CD152 in T cell activation, a blockade assay was performed using rabbit anti-CD152 pAbs. Briefly, 200 µL HKLs (5×10^6^ cells/mL), which had been cultured in DMEM supplemented with 10% FBS, were added to each well of a 96-well plate (VWR). Subsequently, 20 µL heat-activated *A. hydrophila* (5×10^7^ CFU/mL) was also added to each well, and the plate was gently mixed. Thereafter, either the rabbit anti-CD152 pAbs (1 μg/mL) or the rabbit IgG isotype control Abs (1 μg/mL; BioLegend) were added to the cells. Following 6 hours of incubation at 28°C, the cells were harvested and the total RNA was extracted and reverse-transcribed into cDNA. The mRNA expression levels of *CD80/86*, *CD28*, *CD152*, *CD154*, *Lck*, and *CD69* (primers listed in **Table 1**) were determined by qPCR using the 2^−ΔΔCt^ method, with *β-actin* as the internal control

### Statistics Analysis

Statistical analyses were performed using one-way ANOVA with an LSD *post hoc* test, which were performed using the Statistical Product and Service Solution (SPSS) 20.0 software (IBM). A *p*-value of < 0.05 was considered statistically significant.

## Results

### Characterization of the *CD80/86*, *CD28*, and *CD152* Genes in Grass Carp

**Figure 1:**
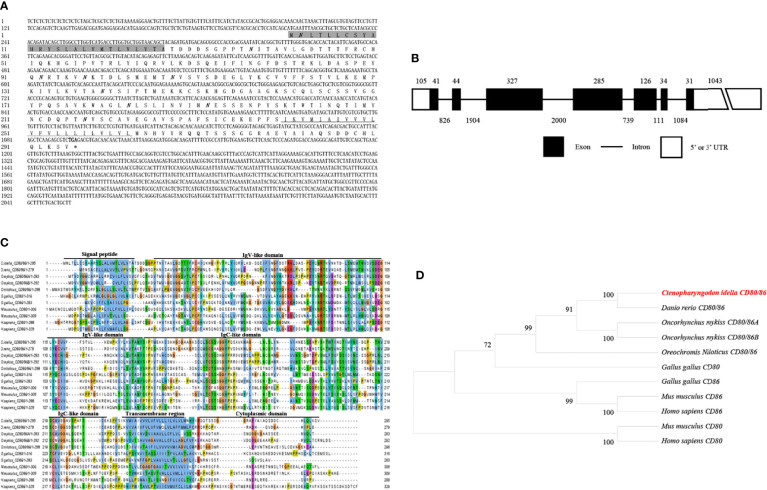
Molecular cloning and bioinformatic analysis of the *CD80/86* gene in grass carp. **(A)** The cDNA and deduced amino acid sequences for CD80/86. The signal peptide is shadowed, and the transmembrane domain is underlined. The stop codon is indicated by the asterisk. The locations of the predicted N-linked glycosylation sites (NGSs) are shown in bold and italic. **(B)** The exon/intron structure of the *CD80/86* gene in grass carp. The rectangles represent the exons, and the lines between them indicate the introns. The black and white areas indicate the coding regions alongside the 5’ or 3’ untranslated regions (UTR), respectively. The size (bp) of the exons and introns are shown by the numbers above and below, respectively. **(C)** Multiple amino acid sequence alignment of CD80/86 homologs. The sequence alignment was conducted using the ClustalX program. The predicted domains of grass carp CD80/86 were marked above the amino acid sequence. **(D)** Phylogenetic analysis of CD80/86 homologs. Protein sequences were used to generate the phylogenetic tree using the neighbor-joining method bootstrapped 1000 times with the MEGA program. The grass carp CD80/86 is shown in red. The GenBank accession numbers for the used sequences are shown below: *Homo sapiens* CD80, NP_005182.1; *H. sapiens* CD86, NP_787058.5; *Mus musculus* CD80, NP_033985.3; *M. musculus* CD86, NP_062261.3; *Gallus gallus* CD80, CAJ18316.1; *G. gallus* CD86, CAJ18297.1; *Danio rerio* CD80/86, NP_001294992.1; *Ctenopharyngodon idella* CD80/86, OL692349; *Oncorhynchus mykiss* CD80/86A, XP_014070713.1; *O. mykiss* CD80/86B, XP_014018862; *Oreochromis niloticus* CD80/86, ATP85027.1.

The *CD80/86*, *CD28*, and *CD152* cDNAs were identified in grass carp and submitted to GenBank with the accession numbers OL692349, UGY70978.1, and UGY70977.1, respectively. The cDNA for the grass carp *CD80/86* gene is 2055 bp in length, which includes an 888 bp open reading frame (ORF) that encodes 295 amino acids with a putative molecular mass of 32.6 kDa (**Figure 1A**). The *CD80/86* gene consists of 7 exons, while the encoded protein consists of a signal peptide, an IgV-like domain, an IgC-like domain, a transmembrane region, and a cytoplasmic domain (**Figures 1B, C**). The multiple amino acid sequence alignment detailed that the CD80/86 protein sequences are conserved throughout vertebrates (**Figure 1C**). The phylogenetic analysis showed that the grass carp CD80/86 was closely clustered with the *D. rerio* CD80/86 (**Figure 1D**). These results combined provide information that the grass carp *CD80/86* gene was successfully cloned in our study.

**Figure 2:**
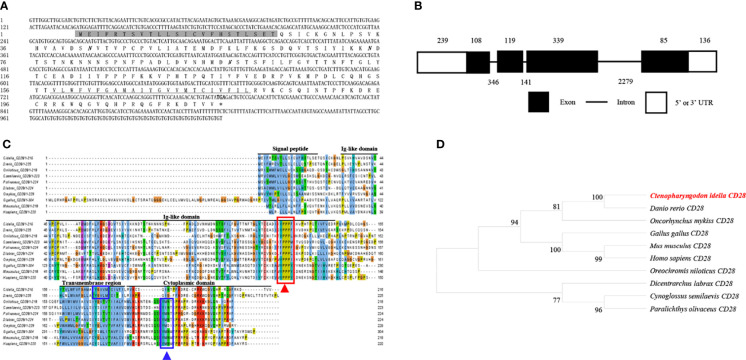
Molecular cloning and bioinformatic analysis of the *CD28* gene in grass carp. **(A)** The cDNA and deduced amino acid sequences of CD28. The signal peptide is shadowed, the transmembrane domain is underlined, and the stop codon is indicated by the asterisk. The predicted NGS locations are shown in bold and italic. **(B)** The exon/intron structure of the *CD28* gene in grass carp. The rectangles represent the exons, and the lines between them indicate the introns. The black and white areas indicate the coding regions and the 5’ or 3’UTRs, respectively. The exon and intron sizes (bp) are shown by the numbers above and below, respectively. **(C)** Multiple amino acid sequence alignment of the CD28 homologs. The sequence alignment was conducted using the ClustalX program. The predicted domains of grass carp CD28 were marked above the amino acid sequence. The conserved binding motifs for binding CD80/86 are indicated with a red rectangle. The conserved tyrosine-based motifs are indicated with blue rectangles. **(D)** Phylogenetic analysis of the CD28 homologs. The phylogenetic tree based on protein sequences was constructed using the neighbor-joining method bootstrapped 1000 times with the MEGA program. The grass carp CD28 is highlighted in red. The GenBank accession numbers of the used sequences are shown below: *H. sapiens*, CAD57003; *M. musculus*, ABH08508.1; *G. gallus*, NP_990642.1; *D. rerio*, XP_001923482.1; *C. Idella*, UGY70978.1; *Cynoglossus semilaevis*, AEM98131; *O. niloticus*, AMQ36815.1; *Dicentrarchus labrax*, AIK66542.1; *Paralichthys olivaceus*, MT019836.1; *O. mykiss*, AAW78853.1.

The cDNA from the grass carp *CD28* gene was 1026 bp long, which included a 651 bp ORF that encoded a 216 amino acid with a putative molecular mass of 24.3 kDa (**Figure 2A**). The *CD28* gene contains 4 exons, and the encoded protein contains a signal peptide, an Ig-like domain, a transmembrane region, and a cytoplasmic domain (**Figures 2B, C**). The multiple amino acid sequence alignment showed that the CD28 protein sequences are conserved through vertebrates (**Figure 2C**). The conserved ^123^YPPPF^127^ motif is responsible for binding the CD80/86 and exists in the Ig-like domain of grass carp CD28. In mammalian CD28, the YXXM motif is present in the cytoplasmic tail, which binds the Src homology 2 domain of p85, an adaptor subunit of PI3K (21). However, in grass carp and zebrafish CD28, the conserved YXXM motif has not been found, which is replaced by a similar motif YXXXM (**Figure 2C**). The phylogenetic analysis illustrated that the grass carp CD28 was closely clustered with the *D. rerio* CD28 (**Figure 2D**). Overall, these results indicate that the grass carp *CD28* gene was successfully cloned in our study.

**Figure 3:**
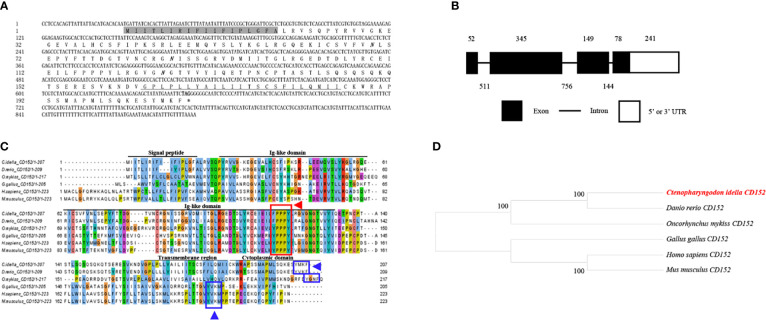
Molecular cloning and bioinformatic analysis of the *CD152* gene in grass carp. **(A)** The cDNA and deduced amino acid sequences of CD152. The signal peptide is shadowed, and the transmembrane domain is underlined. The asterisk indicates the stop codon. The locations of the predicted NGSs are shown in bold and italic. **(B)** The exon/intron structure of the *CD152* gene in grass carp. The rectangles represent the exons, and the lines between them indicate the introns. The black and white areas indicate the coding regions and the 5’ or 3’ UTRs, respectively. The size (bp) of the exons and introns are shown by the numbers above and below, respectively. **(C)** Multiple amino acid sequence alignment of the CD152 homologs. The sequence alignment was conducted using the ClustalX program. The predicted domains of grass carp CD152 were marked above the amino acid sequence. The conserved binding motifs for the CD80/86 binding are highlighted by the red rectangle. The tyrosine-based motifs in the cytoplasmic domain were indicated by blue rectangles. **(D)** Phylogenetic analysis of the CD152 homologs. Protein sequences were used to construct the phylogenetic tree using the neighbor-joining method bootstrapped 1000 times with the MEGA program. The grass carp CD152 is shown in red. The GenBank accession numbers for the used sequences are shown below: *H. sapiens*, NP_005205.2; *M. musculus*, NP_033973.2; *G. gallus*, CAJ86460.1; *D. rerio*, XP_005167576.1; *C. Idella*, UGY70977.1; *O. mykiss*, AAW78854.1.

The cDNA from the grass carp *CD152* gene consisted of 893 bp, 624 bp of which formed the ORF, which encoded 207 amino acids with a putative molecular mass of 23.1 kDa (**Figure 3A**). The *CD152* gene consists of 4 exons and encodes a protein with a signal peptide, an Ig-like domain, a transmembrane region, and a cytoplasmic domain (**Figures 3B, C**). The multiple amino acid sequence alignment demonstrated that the CD152 protein sequences are conserved within vertebrates (**Figure 3C**). The conserved ^115^FPPPY^119^ motif is responsible for binding to CD80/86 and exists in the CD152 Ig-like domain of grass carp. In mammalian and bird CD152, the tyrosine-based motif YVKM in the cytoplasmic domain functions for signal transduction (37, 38). In grass carp and zebrafish CD152, a tyrosine-based motif YXKF also exists in the cytoplasmic domain, which may function as the signal transduction motif. The phylogenetic analysis presented the CD152 in grass carp as closely clustered with the *D. rerio* CD152 (**Figure 3D**). Consequently, these results indicate that the *CD152* gene in grass carp was successfully cloned in our study.

### Expression of the *CD80/86*, *CD28*, and *CD152* Genes in Grass Carp Tissues

**Figure 4:**
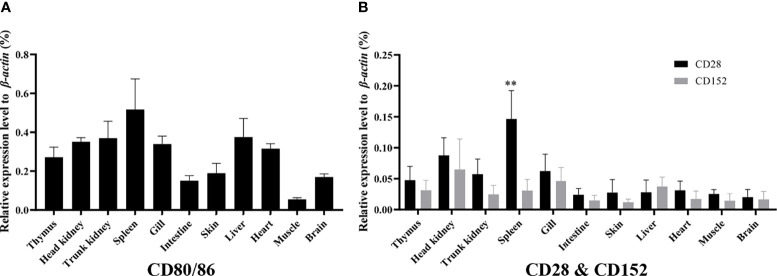
The mRNA expression patterns of the *CD80/86, CD28*, and *CD152* genes in grass carp. **(A)** The tissue mRNA expression pattern of the *CD80/86* gene. **(B)** The tissue mRNA expression patterns of the *CD28* and *CD152* genes. The expression levels were analyzed by qPCR and normalized against the expression of *β-actin* using the 2^−ΔCt^ method. The *p*-value was calculated using ANOVA with the LSD *post hoc* test (**p < 0.01). Data are represented as the mean ± SEM for five individual fish.

The mRNA expression levels of the *CD80/86*, *CD28*, and *CD152* genes were detected in grass carp tissues. The results showed that in grass carp, the *CD80/86*, *CD28*, and *CD152* genes were all constitutively expressed throughout various tissues (**Figures 4A, B**). Indeed, the *CD80/86* gene was highly expressed in the systemic immune tissues, including the spleen, trunk kidney, head kidney, and thymus. Additionally, the *CD80/86* gene was highly expressed in the liver, heart, and gill. Similarly, the *CD28* gene was also highly expressed in the systemic immune tissues and followed the same expression patterns as the *CD80/86* gene. Analysis of the mucosal immune tissues revealed that the *CD28* gene was highly expressed in the gill. Conversely, the *CD152* gene was highly expressed in the head kidney, followed by the gill and liver. Intriguingly, the grass carp *CD28* gene possessed a significant higher expression level than the *CD152* gene only in the spleen.

### Expression of the *CD80/86*, *CD28*, and *CD152* Genes in Grass Carp Leukocyte Subpopulations

**Figure 5:**
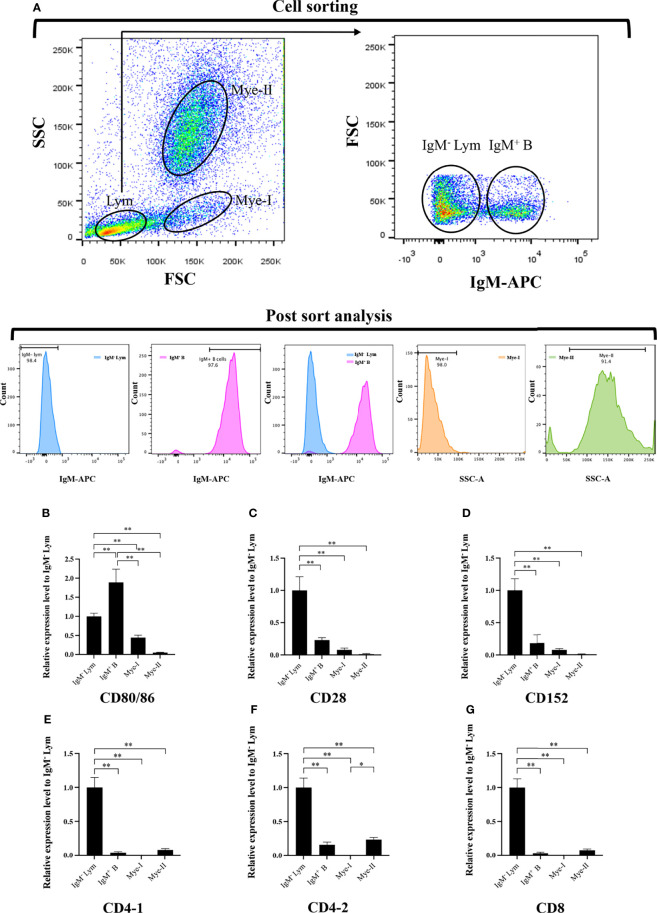
Expression patterns of the grass carp *CD80/86, CD28*, *CD152*, *CD4-1*, *CD4-2*, and *CD8* genes in head kidney leukocyte populations stained with mouse anti-grass carp IgM mAbs. **(A)** Flow cytometry analysis of the HKLs stained with mouse anti-grass carp IgM mAbs and APC-goat anti-mouse IgG Abs. Four different cell populations were sorted: IgM^+^ B cells, IgM^-^ lymphocytes (Lym), myeloid I subset cells (Mye I), and myeloid II subset cells (Mye II). The purity of the sorted cells was analyzed by FACS. **(B–G)** The mRNA expression levels of *CD80/86* **(B)**, *CD28* **(C)**, *CD152* **(D),** *CD4-1* **(E),** *CD4-2* **(F)**, and *CD8* **(G)** genes in the four different cell populations were detected using qPCR. One representative result is represented by the flow cytometry dot plot. The mRNA expression levels were analyzed by qPCR and normalized against *β-actin* using the 2^−ΔCt^ method. The *p* value was calculated using ANOVA with the LSD *post hoc* test (*p < 0.05, **p < 0.01). Data are presented as mean ± SEM (n = 5 fish).

**Figure 6:**
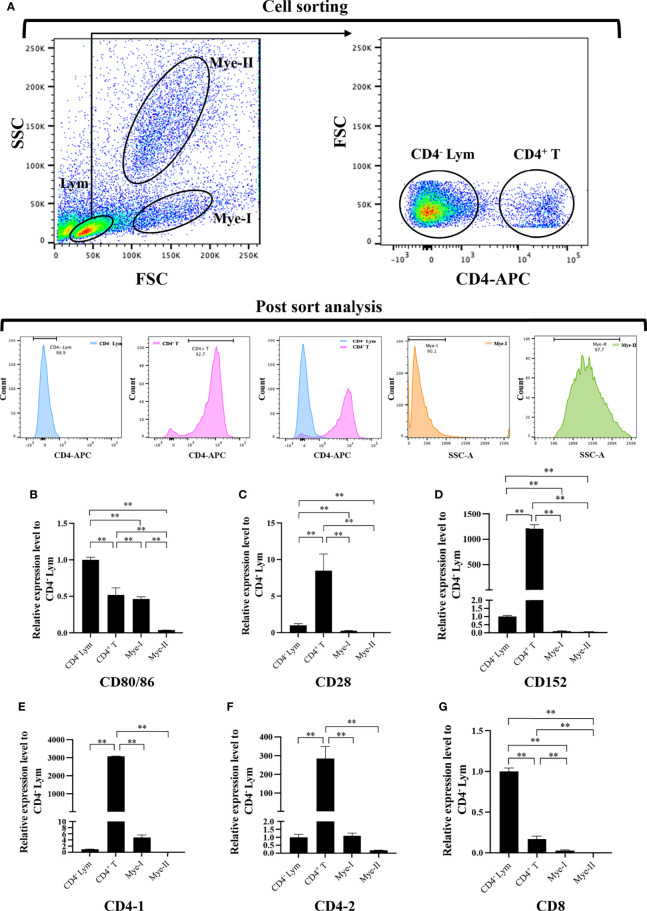
Expression patterns of the grass carp *CD80/86, CD28*, *CD152*, *CD4-1*, *CD4-2*, and *CD8* genes in head kidney leukocyte populations stained with rat anti-carp CD4 mAbs. **(A)** Flow cytometry analysis of the HKLs stained with rat anti-carp CD4 mAbs and APC-goat anti-rat IgG Abs. Four different cell populations were sorted: CD4^+^ T cells, CD4^-^ lymphocytes (Lym), myeloid I subset cells (Mye I), and myeloid II subset cells (Mye II). The purity of the sorted cells was analyzed by FACS. **(B–G)** The mRNA expression levels of *CD80/86* **(B)**, *CD28* **(C)**, *CD152* **(D),** *CD4-1* **(E),** *CD4-2* **(F)**, and *CD8* **(G)** genes in the four different cell populations were detected using qPCR. One representative result is represented by the flow cytometry dot plot. The mRNA expression levels were analyzed by qPCR and normalized against *β-actin* using the 2^−ΔCt^ method. The *p* value was calculated using ANOVA with the LSD *post hoc* test (**p < 0.01). Data are presented as mean ± SEM (n = 3 fish).

**Figure 7:**
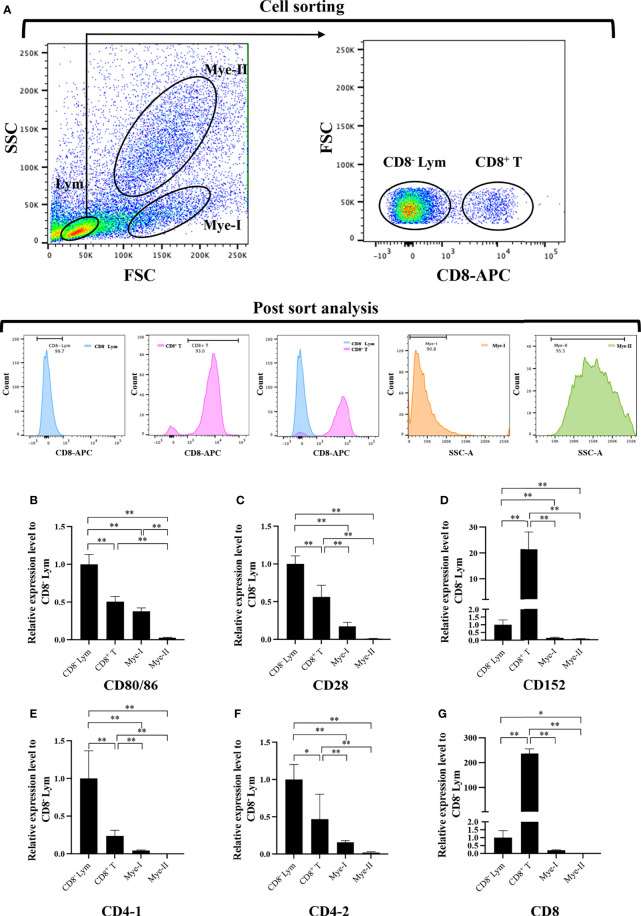
Expression patterns of the grass carp *CD80/86, CD28*, *CD152*, *CD4-1*, *CD4-2*, and *CD8* genes in head kidney leukocyte populations stained with rat anti-carp CD8 mAbs. **(A)** Flow cytometry analysis of the HKLs stained with rat anti-carp CD8 mAbs and APC-goat anti-rat IgG Abs. Four different cell populations were sorted: CD8^+^ T cells, CD8^-^ lymphocytes (Lym), myeloid I subset cells (Mye I), and myeloid II subset cells (Mye II). The purity of the sorted cells was analyzed by FACS. **(B–G)** The mRNA expression levels of *CD80/86* **(B)**, *CD28* **(C)**, *CD152* **(D),** *CD4-1* **(E),** *CD4-2* **(F)**, and *CD8* **(G)** genes in the four different cell populations were detected using qPCR. One representative result is represented by the flow cytometry dot plot. The mRNA expression levels were analyzed by qPCR and normalized against *β-actin* using the 2^−ΔCt^ method. The *p* value was calculated using ANOVA with the LSD *post hoc* test (*p < 0.05, **p < 0.01). Data are presented as mean ± SEM (n = 3 fish).

To further clarify the cells expressing *CD80/86*, *CD28*, and *CD152* genes in grass carp, the IgM^+^ B cells, IgM^-^ lymphocytes, myeloid I subpopulation cells, and myeloid II subpopulation cells were sorted from the head kidney of grass carp. The purity of the sorted cells was checked to be higher than 90% (**Figure 5A**). The results indicated that the *CD80/86* gene was highly expressed in IgM^+^ B cells, which can act as APCs (**Figure 5B**). Contrastingly, both the *CD28* and *CD152* genes were highly expressed in the IgM^-^ lymphocytes (**Figures 5C, D**). Besides, *CD4-1*, *CD4-2*, and *CD8* genes were all highly expressed in the IgM^-^ lymphocytes (**Figures 5E–G**), indicating that the IgM^-^ lymphocytes contain large amounts of CD4^+^ and CD8^+^ T cells. Furthermore, we measured the expression levels of *CD80/86*, *CD28*, and *CD152* genes in CD4^+^ and CD8^+^ T cells (**Figures 6**, **7**). The results indicated that, similar to mammals, grass carp *CD28* and *CD152* were also highly expressed in CD4^+^ and CD8^+^ T cells.

### Grass Carp CD80/86, CD28, and CD152 Express on Cell Surface

**Figure 8:**
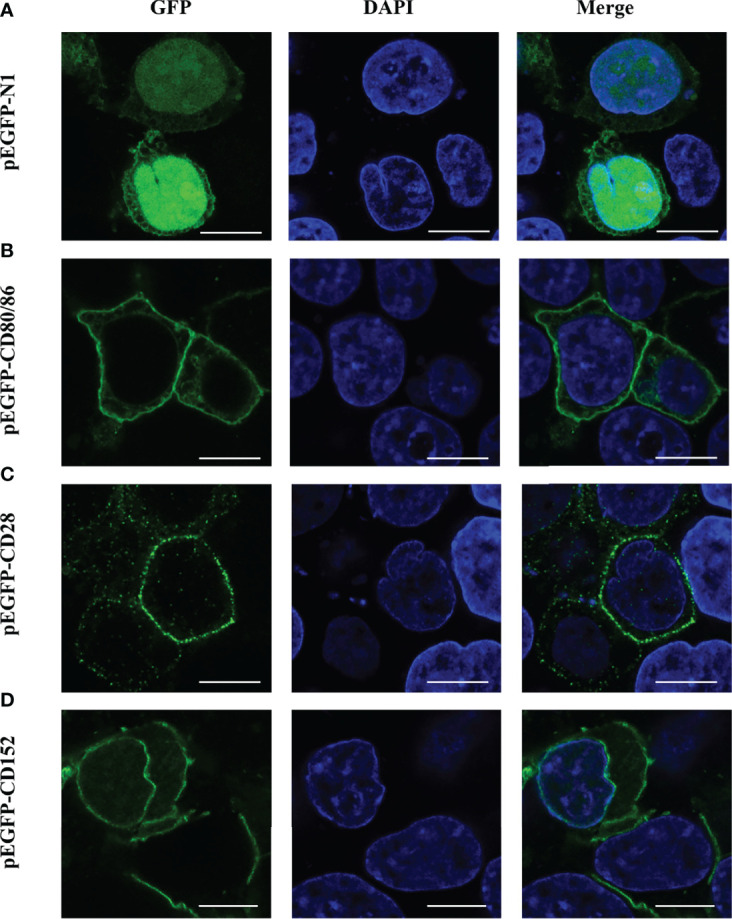
Subcellular localization analyses of CD80/86, CD28, and CD152 in HEK293T cells. **(A)** HEK293T cells were transfected with a pEGFP-N1 plasmid. **(B)** HEK293T cells were transfected with a pEGFP-CD80/86 plasmid. **(C)** HEK293T cells were transfected with a pEGFP-CD28 plasmid. **(D)** HEK293T cells were transfected with a pEGFP-CD152 plasmid. The images were captured using a two-photon laser scanning confocal microscope (Nikon N-storm, Japan). Scale bar = 10 μm.

The subcellular localization of CD80/86, CD28, and CD152 was detected by transfecting either a pEGFP-CD80/86, pEGFP-CD28, or pEGFP-CD152 plasmid into HEK293T cells. Predictably, CD80/86, CD28, and CD152 were all expressed on the cell surface (**Figure 8**). Yet, the GFP protein was distributed within the cytoplasm and nucleus, indicating that the signals for CD80/86, CD28, and CD152 were cell surface-specific.

### The Grass Carp CD80/86 can Bind to CD28 and CD152

**Figure 9:**
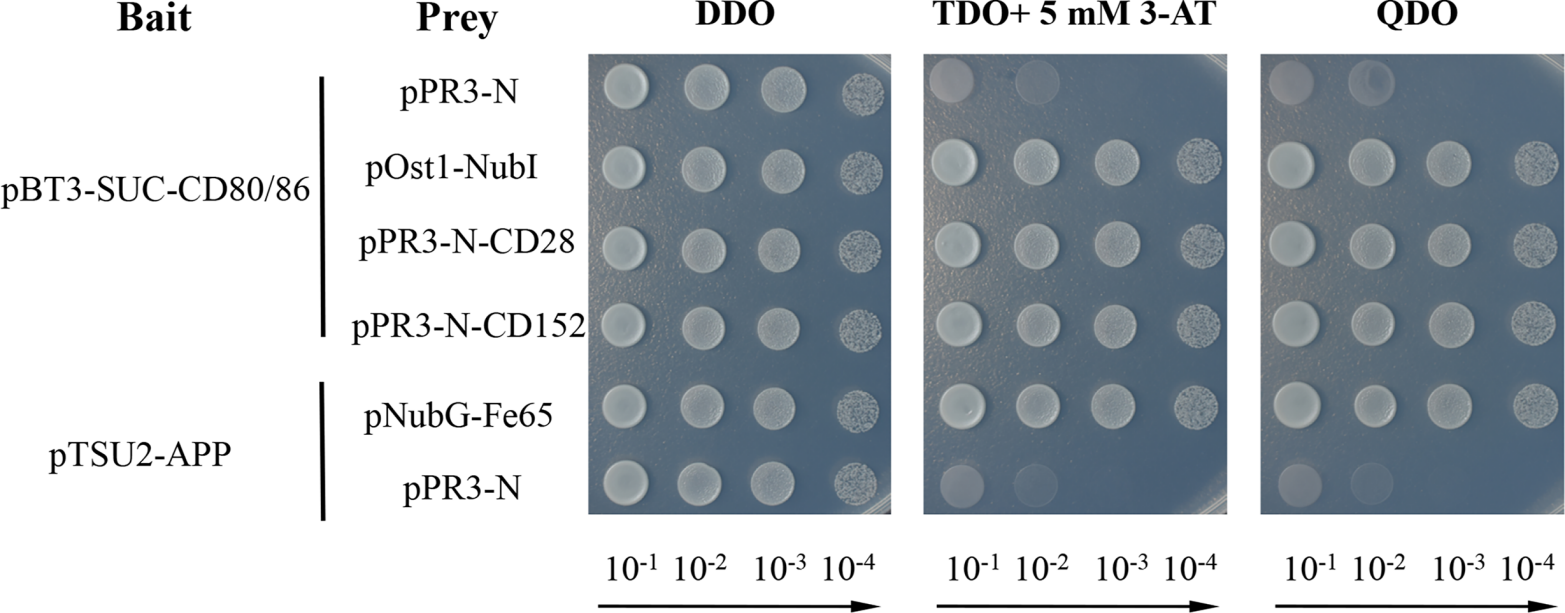
Yeast two-hybrid assay of the interaction between CD80/86 and either CD28 or CD152. The pPR3-N and pBT3-SUC-CD80/86 plasmids were co-transformed into NMY51 yeast cells as the self-activation control. The pOst1-NubI and pBT3-SUC-CD80/86 plasmids were co-transformed into NMY51 yeast cells as a functional assay control. The two experimental groups, the pBT3-SUC-CD80/86 and pPR3-N-CD28 plasmids, alongside the pBT3-SUC-CD80/86 and pPR3-N-CD152 plasmids, were both co-transformed into the NMY51 yeast cells. The pNubG-Fe65 and pTSU2-APP plasmids were used as a positive control in NMY51 yeast cells, while pPR3-N and pTSU2-APP acted as the negative control. Following co-transformation, the yeast cells were inoculated on plates with double dropout medium (DDO: SD/−Leu/−Trp), triple dropout medium/3-AT (TDO/3-AT: SD/−Leu/−Trp/−His, supplemented with 5 mM 3-Aminotriazole), and quadruple dropout medium (QDO: SD/−Leu/−Trp/−His/−Ade). The successful plasmid transfection was indicated by positive yeast clones on the DDO plates. The presence of clones on the TDO/3-AT plates signified the successful expression of the *His3* reporter gene, while the presence of clones on the QDO denoted the successful expression of the *Ade2* reporter gene. These results showed that CD80/86 can interact with both CD28 and CD152 in the yeast two-hybrid system.

A membrane two-hybrid assay was performed in yeast to detect the binding activity of the grass carp CD80/86 to CD28 and CD152. The yeast cells co-transformed with plasmids pBT3-SUC-CD80/86 and pPR3-N could grow on the DDO medium but not on the TDO and QDO media, suggesting that CD80/86 itself had no transcriptional activity. Further study revealed that the grass carp CD80/86 could bind to CD28 since the yeast cells co-transformed with plasmids pBT3-SUC-CD80/86 and pPR3-N-CD28 could grow on the TDO and QDO media (**Figure 9**). Moreover, the grass carp CD80/86 could also bind to CD152, which was exhibited by the yeast cells co-transformed with the plasmids pBT3-SUC-CD80/86 and pPR3-N-CD152 growing on both the TDO and QDO media. Conversely, the negative control, consisting of yeast cells co-transformed with the pBT3-SUC-CD80/86 and pPR3-N plasmids, could not grow on the TDO and QDO media. Therefore, the grass carp CD80/86 binding activities to CD28 and CD152 were specific.

### The Dynamic Expression of the Grass Carp *CD80/86*, *CD28*, and *CD152* Genes Following Infection

**Figure 10:**
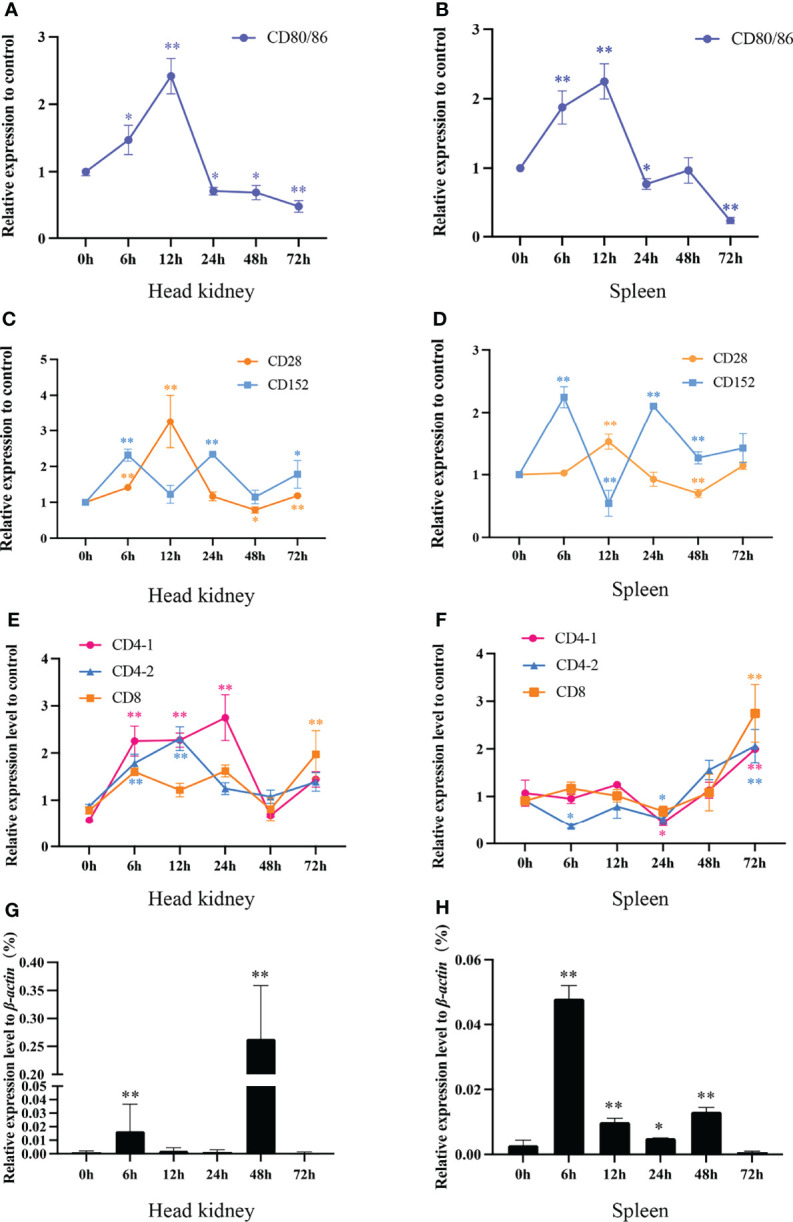
The mRNA expression levels of *CD80/86*, *CD28*, *CD152*, *CD4-1*, *CD4-2*, and *CD8* genes in grass carp head kidney and spleen following the *A. hydrophila* infection. **(A)** The mRNA expression level of *CD80/86* in the head kidney. **(B)** The mRNA expression level of *CD80/86* in the spleen. **(C)** The mRNA expression levels of *CD28* and *CD152* in the head kidney. **(D)** The mRNA expression levels of *CD28* and *CD152* in the spleen. **(E)** The mRNA expression levels of *CD4-1*, *CD4-2*, and *CD8* in the head kidney. **(F)** The mRNA expression levels of *CD4-1*, *CD4-2*, and *CD8* in the spleen. **(G)** The expression level of 16s RNA in the head kidney. **(H)** The expression level of 16s RNA in the spleen. The fold changes of *CD80/86*, *CD28*, *CD152*, *CD4-1*, *CD4-2*, and *CD8* were calculated by comparing the stimulated group with the control group (defined as 1) using the 2^−ΔΔCt^ method. The expression level of 16s RNA was analyzed by qPCR and normalized against grass carp *β-actin* using the 2^−ΔCt^ method. The data represent the mean ± SEM for 4 individual fish. The *p*-value was calculated by one-way ANOVA with the LSD *post hoc* test (*p < 0.05, **p < 0.01).

To gain further insights into the interaction features of CD80/86, CD28, and CD152 in grass carp, the immune responses of these genes were detected following the onset of a bacterial infection. The data show that the mRNA expression was rapidly induced for both *CD80/86* and *CD28* in the head kidney and spleen in response to the *A. hydrophila* infection (**Figures 10A–D**). The expression levels peaked at 12 hpi and then decreased back to the control levels at 24 hpi. Overall, the expression levels of *CD80/86* and *CD28* exhibited a rise followed by a decline over the 3-day experimental period. However, the mRNA expression of *CD152* displayed a cyclical pattern in both the head kidney and spleen after an *A. hydrophila* infection; whereby, it initially rapidly increased at 6 hpi, decreased at 12 hpi, before again increasing at 24 hpi, and then decreased again at 48 hpi.

In addition, we tested the expression levels of *CD4-1*, *CD4-2*, and *CD8* genes in both head kidney and spleen (**Figures 10E, F**). In head kidney, the expression levels of *CD4-1*, *CD4-2*, and *CD8* upregulated rapidly after the infection, decreased at 48 hpi, then again increasing at 72 hpi. Unlike those in head kidney, the expression levels of *CD4-1*, *CD4-2*, and *CD8* in spleen all upregulated rapidly at 24 hpi.

After *A. hydrophila* infection, the bacterial load in head kidney and spleen was determined by qPCR (**Figures 10G, H**). The results showed that both in head kidney and spleen, the bacterial load rapidly increased at 6 hpi, decreased at 12- and 24-hpi, before again increasing at 48 hpi, and then decreased again at 72 hpi.

### Rabbit Anti-Grass Carp CD152 pAbs Can Specifically Recognize CD152

**Figure 11:**
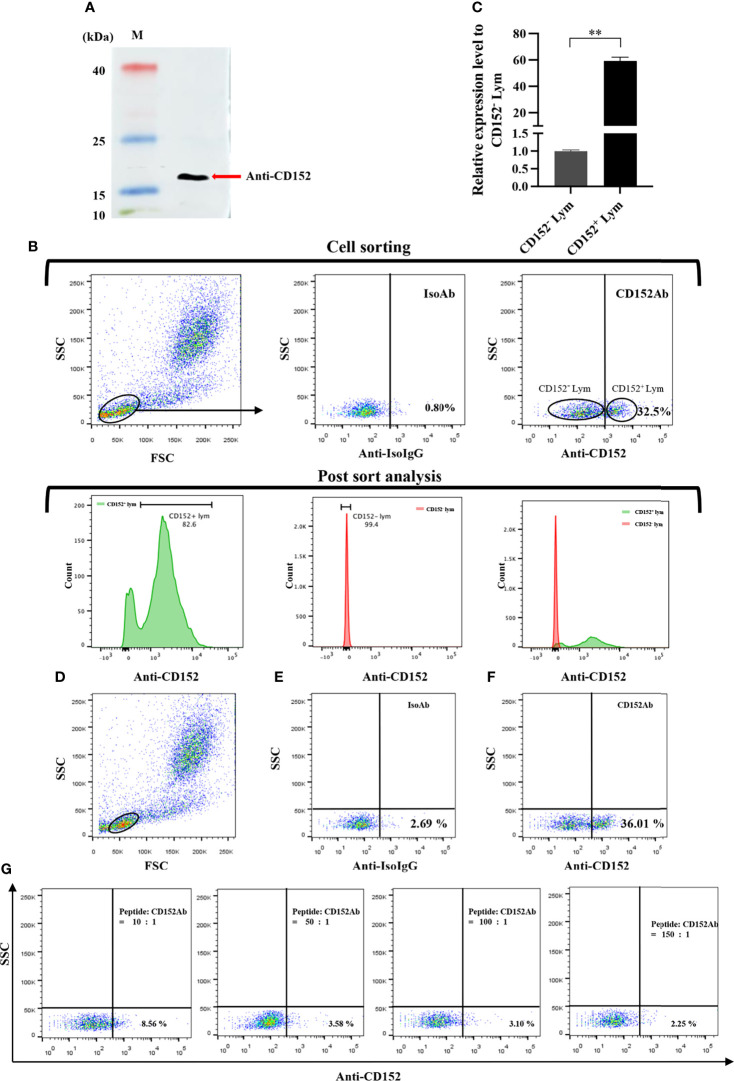
Investigating the specificity of the rabbit anti-grass carp CD152 pAbs. **(A)** Western blot analysis of the rabbit anti-CD152 pAbs. The rabbit anti-CD152 pAbs can specifically recognize the CD152 protein in HKLs. **(B)** FACS analysis of the CD152^+^ Lym and CD152^-^ Lym. The HKLs were stained with the rabbit anti-CD152 pAbs, prior to sorting the CD152^+^ Lym and CD152^-^ Lym cell populations and performing RNA isolation and cDNA synthesis. The purity of sorted cells was analyzed by flow cytometry. **(C)** The mRNA expression level of *CD152* in CD152^+^ Lym and CD152^-^ Lym. **(D)** The gating strategy for the flow cytometric analysis of HKLs stained with rabbit anti-CD152 pAbs. The lymphocytes (Lym) were gated to provide further analysis of the CD152^+^ cells. **(E)** The gated lymphocytes in HKLs were stained with the rabbit IgG isotype control Abs. **(F)** The gated lymphocytes in HKLs were stained with the rabbit anti-CD152 pAbs. **(G)** The rabbit anti-CD152 pAbs were preincubated with the peptide at different molar ratios for 45 minutes, before being applied to stain the HKLs for flow cytometry. The mRNA expression level of *CD152* was analyzed by qPCR and normalized against the expression of *β-actin* using the 2^−ΔCt^ method. Fold changes were calculated by comparing the CD152^+^ Lym with the CD152^-^ Lym (defined as 1). The data in **(C)** are presented as mean ± SEM (n = 5 fish). One representative result is shown in **(D–G)**. The *p*-value was calculated by one-way ANOVA with the LSD *post hoc* test (**p < 0.01).

We produced and purified rabbit anti-grass carp CD152 pAbs using an antigenic peptide. The result of western blot showed that the rabbit anti-grass carp CD152 pAbs could specifically recognize the CD152 protein in HKLs (**Figure 11A**). To further confirm the specificity of the pAbs, FACS was employed to sort the CD152^+^ Lym and CD152^-^ Lym (**Figure 11B**). Data generated from qPCR demonstrated that the *CD152* mRNA was highly expressed in CD152^+^ Lym compared to the CD152^-^ Lym (**Figure 11C**). The blocking experiment also confirmed that the pAbs were specific for grass carp CD152 since the antigenic peptide could block the pAbs binding to lymphocytes (**Figures 11D–G**). These data indicate that the rabbit anti-grass carp CD152 pAbs can specifically recognize CD152.

### Role of CD152 in T Cell Activation

**Figure 12:**
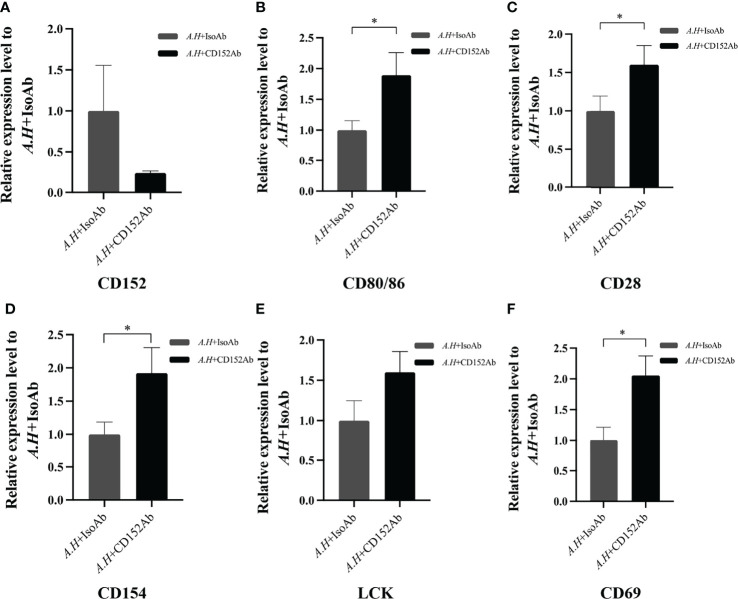
Blocking CD152 by anti-CD152 pAbs on HKLs stimulated by *A. hydrophila*. The HKLs supplemented with the rabbit IgG isotype Abs were designated as the control group, and the HKLs supplemented with rabbit anti-grass carp CD152 pAbs as the experimental group. Six hours post-stimulation, the HKLs were harvested, the RNA isolated and the cDNA synthesized. **(A)** The mRNA expression level of *CD152*. **(B)** The mRNA expression level of *CD80/86*. **(C)** The mRNA expression level of *CD28.* **(D)** The mRNA expression level of *CD154*. **(E)** The mRNA expression level of *LCK*. **(F)** The mRNA expression level of *CD69*. The mRNA expression levels were analyzed by qPCR using the 2^−ΔΔCt^ method, with *β-actin* as the internal control. Data are presented as mean ± SEM (n = 5 fish). The *p*-value was calculated by one-way ANOVA with the LSD *post hoc* test (*p < 0.05).

Subsequently to evaluate the roles of CD152 in T cell activation after an *A. hydrophila* stimulation, a blockade assay was performed using the rabbit anti-grass carp CD152 pAbs. The results showed that the rabbit anti-grass carp CD152 pAbs inhibited the expression of *CD152* in HKLs (**Figure 12A**). This indicates that the rabbit anti-grass carp CD152 pAbs can bind to the CD152 on HKLs. Interestingly, the expression levels of *CD80/86* and *CD28* as well as the hallmark genes related to T cell activation, such as *Lck*, *CD154* (*CD40L*), and *CD69*, were higher in the HKLs incubated with the rabbit anti-grass carp CD152 pAbs compared to those incubated with isotype control Abs (**Figures 12B–F**), suggesting that the grass carp CD152 is a negative receptor for T cell activation.

## Discussion

T cells play vital roles in the adaptive immunity of mammals. The effective activation of T cells requires the co-stimulatory signals provided by CD80 and CD86, which occur through binding to CD28 and CD152 on T cells (39–42). However, since only a primordial CD80/86 molecule exists in the teleost species (except salmonids) (11), the mechanism relating to how the primordial CD80/86 interacts with its CD28 and CD152 receptors to activate T cells remains unknown. Thus, to elucidate the mechanism, we successfully cloned and identified the grass carp *CD80/86*, *CD28*, and *CD152* genes in this study. The grass carp *CD80/86* gene consists of 7 exons and 6 introns. Previous studies revealed that the zebrafish *CD80/86* gene consists of 6 exons and 5 introns (12), while the rainbow trout *CD80/86* gene consists of 8 exons and 7 introns (43). These differences in structure indicate that the exon/intron organization of the *CD80/86* gene is not conserved across teleost species. Contrastingly, the *CD28* and *CD152* gene structures are consistent across teleost species, whereby the *CD28* and *CD152* genes in grass carp and other known teleost fish are all comprised of 4 exons and 3 introns. In mammalian CD28, the YXXM motif in the cytoplasmic tail were confirmed for binding the Src homology 2 domain of p85, an adaptor subunit of PI3K (21). However, in grass carp and zebrafish CD28, the conserved YXXM motif has not been found, which is replaced by a similar motif YXXXM. Moreover, the existence of the tyrosine-based motif YXKF in the cytoplasmic tail of grass carp and zebrafish CD152 indicates that CD152 may have a conserved signal transduction pattern in teleost fish. Overall, the protein sequences and domain compositions of CD80/86, CD28, and CD152 are conserved within vertebrates, indicating the presence of functional conservation within these molecules throughout evolution.

Previous studies have proven that the mammalian CD28 is expressed on both resting and activated T cells, but CD152 is expressed only on activated T cells (44–46). In contrast to that in humans, grass carp *CD80/86*, *CD28*, and *CD152* were shown to be constitutively expressed in various tissues, particularly the immune tissues. The *CD28* expression level was found to be higher than the *CD152* in most of the analyzed grass carp tissues. Notably, in the grass carp liver, the expression level of *CD152* was even higher than that of *CD28*. Similar results have been reported in rainbow trout and sea bass (16, 18). The distinct expression patterns of *CD28* and *CD152* in teleost fish imply that the costimulatory signals for T cell activation in teleost fish may be different from those in mammals. Moreover, we found that *CD80/86* was highly expressed in IgM^+^ B cells, which can act as APCs in teleost fish (12). Further, *CD28* and *CD152* were highly expressed in CD4^+^ and CD8^+^ T cells. Therefore, similar to mammals, the teleost *CD80/86* are also expressed by APCs, while *CD28* and *CD152* are expressed by T cells. Importantly, the grass carp CD80/86 was revealed to bind to both the CD28 and CD152 in a yeast two-hybrid assay. This result was further confirmed by the demonstration that CD80/86, CD28, and CD152 in grass carp are all membrane molecules expressed on the cell surface, which is necessary for the signaling between the costimulatory molecules. Moreover, the existence of the conserved B7 binding sites in the grass carp CD28 (YPPPF) and CD152 (FPPPY) further supports the conclusion that the binding activities of CD80/86 to CD28 and CD152 are specific (18–21).

During T cell activation in mammals, CD86 mainly binds to CD28 to augment T cell activation, while CD80 primarily binds to CD152 to inhibit the over-activation of T cells (47–49). In this study, we found that following the bacterial stimulation, the *CD80/86* expression dramatically increased in head kidney and spleen. However, the expression of *CD28* and *CD152* exhibited contradictory expressions, suggesting that CD28 and CD152 perform opposing roles in the activation of teleost T cells. Similar mRNA expression patterns were also found in sea bass and rainbow trout after stimulation (16, 18). These further indicated that CD28 and CD152 may play contrary roles in the activation of T cells in other teleost fish. It is noteworthy that, after bacterial infection, the expression of grass carp CD28 and CD152 showed negative and positive correlation respectively with the bacterial load in head kidney and spleen, suggesting that CD28 and CD152 play important opposing roles in the immune system of grass carp *in vivo*. Furthermore, we found that similar to mammalian CD152, the grass carp CD152 negatively regulated T cell activation, for the expression of T cell markers (*CD154*, *LCK*, and *CD69*) was promoted by anti-CD152 pAbs after the bacterial stimulation. Taken together, these results suggest that a moderate activation of T cells in teleost fish can be achieved through the dynamic expression of the stimulatory receptor CD28 and the inhibitory receptor CD152. However, the mechanism requires further elucidation such as the binding affinity of CD28 and CD152 to CD80/86.

In summary, this study characterized CD80/86, CD28, and CD152 in grass carp, the homologs of which are conserved in vertebrates. Moreover, the interaction feature between primordial CD80/86 and its receptors CD28 and CD152 was illustrated in a teleost fish. Overall, this study increases the understanding of the evolution of costimulatory signals in vertebrates.

## Data Availability Statement

The datasets presented in this study can be found in online repositories. The names of the repository/repositories and accession number(s) can be found in the article/**Supplementary Material**.

## Ethics Statement

The animal study was reviewed and approved by ethical guidelines of Huazhong Agricultural University.

## Author Contributions

T-ZL performed most of the experiments, analyzed most of the data, and wrote the preliminary manuscript. XL and Z-YM participated in the infection experiments of grass carp by *A. hydrophila.* C-SW searched the *CD80/86*, *CD28*, and *CD152* genes in the grass carp genome. YW helped with the sampling and the qPCR. Y-AZ helped with experiment design and revised the manuscript. X-JZ designed the research, analyzed some of the data, and revised the manuscript. All authors contributed to the article and approved the submitted version.

## Funding

This work was supported by the National Natural Science Foundation of China (31972824, 31930114), the Open Project of Guangdong Provincial Key Laboratory of Pathogenic Biology and Epidemiology for Aquatic Economic Animals (PBEA2021ZD02), and the Fundamental Research Funds for the Central Universities (2662018QD053).

## Conflict of Interest

The authors declare that the research was conducted in the absence of any commercial or financial relationships that could be construed as a potential conflict of interest.

## Publisher’s Note

All claims expressed in this article are solely those of the authors and do not necessarily represent those of their affiliated organizations, or those of the publisher, the editors and the reviewers. Any product that may be evaluated in this article, or claim that may be made by its manufacturer, is not guaranteed or endorsed by the publisher.

## Abbreviations

Ab, antibody; APC, antigen-presenting cell; CDS, coding sequence; DDO, double dropout medium (SD/–Leu/–Trp); FSC, forward scatter; HKL, head kidney leukocyte; Lym, lymphocyte; mAb, monoclonal antibody; Mye, myeloid cell; Mye-I, myeloid cell subset-I; Mye-II, myeloid cell subset-II; ORF, open reading frame; pAb, polyclonal antibody; QDO, quadruple dropout medium (SD/–Leu/–Trp/–His/–Ade); qPCR, quantitative real-time PCR; SSC, side scatter; TDO, triple dropout medium (SD/–Leu/–Trp/–His); UTR, untranslated region; 3-AT, 3-aminotriazole.
