# Supplementary material for: Molecular and Functional Analyses of the Primordial Costimulatory Molecule CD80/86 and Its Receptors CD28 and CD152 (CTLA-4) in a Teleost Fish
Source: Front Immunol. 2022 Jun 16;13:885005. doi: 10.3389/fimmu.2022.885005 (PMC9245511; doi:10.3389/fimmu.2022.885005)
Supplement: Supplementary file 1 [file DataSheet_1.docx]

Supplementary Material

# Supplementary Figure

**SSC**

**FSC**

**SSC**

**SSC**

**SSC**

**Anti-CD152**

**Anti-CD152**

**A**

**B**

**C**

**D**

**Anti-CD152**

**Supplementary Figure 1.** The reactivity of the rabbit anti-grass carp CD152 pAbs on the leukocyte subpopulations. (A) Flow cytometry analysis of the HKLs stained with rabbit anti-grass carp CD152 pAbs and FITC-goat anti-rabbit IgG pAbs. Three different cell populations were gated. (B) The reactivity of the rabbit anti-grass carp CD152 pAbs on myeloid II subpopulation cells. (C) The reactivity of the rabbit anti-grass carp CD152 pAbs on myeloid I subpopulation cells. (D) The reactivity of the rabbit anti-grass carp CD152 pAbs on lymphocytes.
